# Sickness Absence in the Private Sector of Greece: Comparing Shipyard Industry and National Insurance Data

**DOI:** 10.3390/ijerph9041171

**Published:** 2012-04-02

**Authors:** Evangelos C. Alexopoulos, Georgios Merekoulias, Dimitra Tanagra, Eleni C. Konstantinou, Efi Mikelatou, Eleni Jelastopulu

**Affiliations:** 1 Public Health Department, Medical School, University of Patras, 26504, Greece; Email: gmerek@upatras.gr (G.M.); dimtanagra@gmail.com (D.T.); jelasto@upatras.gr (E.J.); 2 Occupational Health Department, Onassis Cardiac Surgery Center, Athens, 17674, Greece; 3 Faculty of Health and Caring Professions, Technological Educational Institute of Athens, 12210, Greece; Email: ekonstan@teiath.gr (E.C.K.); efimikelatou@yahoo.gr (E.M.)

**Keywords:** sickness absence, sick leave, occupational health, social insurance, employment, compensation, shipyard, industry, Greece

## Abstract

Approximately 3% of employees are absent from work due to illness daily in Europe, while in some countries sickness absence exceeds 20 days per year. Based on a limited body of reliable studies, Greek employees in the private sector seem to be absent far less frequently (<5 days/year) compared to most of the industrialized world. The aim of this study was to estimate the levels of sickness absence in the private sector in Greece, using shipyard and national insurance data. Detailed data on absenteeism of employees in a large shipyard company during the period 1999–2006 were utilized. National data on compensated days due to sickness absence concerning all employees (around 2 million) insured by the Social Insurance Institute (IKA, the largest insurance scheme in Greece) were retrieved from the Institute’s annual statistical reports for the period 1987–2006. Sick-leave days per employee and sick-leave rate (%) were calculated, among other indicators. In the shipyard cohort, the employment time loss due to sick leave was 1%. The mean number of sick-leave days per employee in shipyards ranged between 4.6 and 8.7 and sick-leave rate (sickness absenteeism rate) varied among 2% and 3.7%. The corresponding indicators for IKA were estimated between 5 and 6.3 sick-leave days per insured employee (median 5.8), and 2.14–2.72% (median 2.49%), respectively. Short sick-leave spells (<4 days) may account at least for the 25% of the total number of sick-leave days, currently not recorded in national statistics. The level of sickness absence in the private sector in Greece was found to be higher than the suggested by previous reports and international comparative studies, but still remains one of the lowest in the industrialized world. In the 20-years national data, the results also showed a 7-year wave in sickness absence indexes (a decrease during the period 1991–1997 and an increase in 1998–2004) combined with a small yet significant decline as a general trend. These observations deserve detailed monitoring and could only partly be attributed to the compensation and unemployment rates in Greece so other possible reasons should be explored.

## 1. Introduction

Monitoring of sickness absences is an essential part of occupational health care. Even though sickness absence is not a simple function of ill health, and includes psychosocial factors and coping behaviors, it still remains a valuable tool to assess the impact of disease or other factors on a worker’s capacity and health status [1,2]. The relationship between occupational health risks and sickness absence has been well documented [3,4,5], supporting the need to monitor and evaluate sick-leave spells for both prognosis and early intervention. Absenteeism as a phenomenon has been the focus of research for several decades [6], with an increasing body of literature over the years [7], establishing the need for monitoring which is performed mainly by national statistical services and social security organizations across countries [8,9,10,11].

The definition of absenteeism has been an issue of debate for several conferences of the International Labor Organization (ILO) [12], troubling researchers in the field to the point that today this makes cross-country comparisons difficult. ILO defines absence due to illness as the absence from work, usually of short duration, that may be work related or not, which is attributed by the employee to illness or injury and is accepted by the employer as such [13]. The European Council and most organizations calculate on a yearly basis the compensated working days per employee (days for which employees receive salary compensation) lost due to sickness or injury [9,10,11,12]. Other reasons such as vacations, maternity and paternity leave, educational leave or strikes can also contribute to absenteeism, but are usually not monitored [10,12,14].

Several indicators have been used to monitor absenteeism [15], like: (i) Absence from work; (ii) Frequency of occurrence of absence; (iii) Cumulative incidence (individual frequency); and (iv) Absenteeism rate (%). The International Social Security Association (ISSA) in order to facilitate international comparisons, proposed in 1981 to use absenteeism rate, which express the percentage of working time lost, as a monitor tool since it takes into account the differences in working time per country or occupation [11,16].

International comparative studies are few, with several methodological problems mainly because of lack of widely accepted standard measures of absenteeism, and trends in absenteeism have not been explored. The CESifo DICE Report [17] is such an effort, but the data extracted by OECD (Organization for Economic Co-Operation and Development) Health [9] and WHO Health for all databases [10] were incomplete, allowing several arguments. In the CESifo DICEreport [17] Greece was ranked in the most favorable position for the period 1996–2005, with an average of 4.9 sick leave days per employee per year, while Germany, UK and Netherlands had shown 16.7, 7.6 and 5.1, respectively. In the Fourth European Working Conditions Survey [18], Greek employees reported both the most unfavorable working conditions but the lowest sickness absence (<4 days per year).

The IMF staff paper on work absence in Europe [19] for the period 1983–2003, has shown that the percentage of employees who were absent daily was <0.5% in Greece, showing a declining trend, whereas in most European countries the percentage exceeded 2.5%. Similarly in the European Labour Force Survey the absenteeism rate for the period 2004–2006 for Greece does not exceed 0.2%, while for most countries it exceeded 1.5% [20]. These studies used available data from Eurostat, OECD Health database and WHO Health for all databases and despite some inconsistencies, Greek employees seem to be less frequently absent compared to the most industrialized countries worldwide [7]. 

Many of the differences on sickness absences across countries may be attributed to the respective sickness insurance policies. In the Greek private sector, a worker on sick leave due to illness will be compensated by the company with 50% of his wage; occupational injuries (accidents) are fully compensated. In fact this lasts till the third day of a sick-leave spell, and afterwards the employee will be paid (50%) by the social insurance coverage scheme. Employees can get sickness benefits for up to one year and in some cases two years, before getting a disability pension, or even be fired if the employer or the insurance company argues that the employee is not willing to follow instructions to improve his health. In general an employee is entitled of sickness benefits for as many months as the years of employment completed. Sick leaves up to 2 days are commonly accepted by employee’s report of sickness (not exceeding 3–4 different spells per year). For sick-leave spells longer than 3 days a physician’s certificate is required and if the sick-leave spell is longer than 10 days it has to be validated by the insurance medical committee.

In general, there are relatively few comparative studies on sickness absence, with several limitations (variations in the average annual working days; incompleteness in the monitoring networks and in the methodologies used) and it is considered that sickness absence has not been the subject of a systematic investigation in Greece so far [21]. Based on these, we undertook this research in order to estimate the levels of sickness absence in the private sector in Greece, using shipyard and national insurance data.

## *2*. Results and Discussion

Table 1 presents various indicators of the insured employees at the Social Insurance Institute (IKA) for the period 1987 to 2006. Total compensated sick-leave days reached 7.65 million at 2004. A steady increase in the insured population by 1% per year was monitored since 1987. In the 20-years national data, the results also showed a 7-year wave in sickness absence indexes (a decrease during the period 1991–1997 and an increase in 1998–2004) combined with a small yet significant decline as a general trend. The annual sick-leave rate during the last decade hardly exceeded 2.5% and the mean sick leave duration per employee was well below 6 days (Table 1). 

**Table 1:** Sickness absence in IKA insured employees (private sector) in Greece from 1987 to 2006.

Year	Number of employees insured (in thousands)	Compensated days of sickness absence (excluding gestation)	Total sick-leave days per employee corrected ^1^	Sick-leave rate (%)
1987	1744	6,907,071	6.08	2.62
1988	1766	7,050,071	6.12	2.64
1989	1795	7,293,236	6.22	2.68
1990	1812	7,503,286	6.32	2.72
1991	1831	7,288,640	6.11	2.63
1992	1849	7,112,831	5.93	2.56
1993	1861	7,116,797	5.90	2.54
1994	1874	6,987,209	5.77	2.49
1995	1884	6,611,503	5.48	2.36
1996	1889	6,155,086	5.14	2.22
1997	1902	5,956,481	4.97	2.14
1998	1907	6,153,744	5.10	2.20
1999	1935	6,552,417	5.31	2.29
2000	1941	6,882,306	5.53	2.38
2001	1949	7,143,751	5.69	2.45
2002	1952	7,303,804	5.79	2.49
2003	1956	7,380,596	5.83	2.51
2004	1961	7,644,917	6.00	2.58
2005	1965	7,153,368	5.65	2.44
2006	2031	6,823,353	5.28	2.28

^1^ measures corrected for both the not recorded short sick-leave spells (<4 days) and the not compensated (first 3) days.

Table 2 and Table 3 present the characteristics and main results from the shipyard employees (92% males; mean age 38 ± 10 years) for the period 1999–2006. Approximately 60% of the employees who were absent at least once appear to have short term sick leaves. Out of total spells every year 75% were short term, representing approximately 25% of the total sick-leave days. The percentage of total employees with at least one sick-leave spell per year increased from 36% in 1999 to 52% in 2006 (Table 2).

During the 8-year study period the duration of sickness absence per employee ranged between 4.6 and 8.7 days/year, with an increasing tendency, while the mean duration of each sick-leave spell ranged between 5.9 and 8.6 days, demonstrating a declining trend. Around 1% of total employees were absent due to illness every day (Table 3). Frequency of sickness absence occurrence along with its cumulative incidence were steadily rising, indicating that over the years more employees tend to be more frequently absent due to illness. Sick leave rate ranged between 1.42 and 2.68% with an increasing trend after 2001.

Shipyards data have shown that an average of 6.2 days lost per employee due to sick-leave compared to the estimated 5.9 days of IKA employees. Our data confirm that Greece has one of the lowest sickness absence rates in the European Union [18]. However the level of sickness absence seems to be higher than the estimated rates in previous reports [17,19,20,22] and this may attributed in the more frequent rather than longer sick-leave spells. 

**Table 2:** Sickness absence in a shipyard industry in Greece in 1999−2006, presented by year using different measures of sickness absence.

Year	No of employees with at least one sick-leave spell		No of employees with short sick-leave spells (<4 days)			Total sick-leave spells	Short sick-leave spells (<4 days)		Total number of sick leave days	Total number of sick leave days in short sick-leave spells (<4 days)	
	*n*	% of total employees	*n*	% of total employees	% of employees with at least one sick-leave spell	*N*	*n*	% of total sick-leave spells	*n*	*n*	% of total number of all sick-leave days
1999	716	36	Na	Na		1208	Na	Na	10,386	Na	Na
2000	767	38	Na	Na		1188	Na	Na	10,255	Na	Na
2001	865	41	Na	Na		1121	Na	Na	9698	Na	Na
2002	794	47	496	29	62	1632	1236	76	10,930	2534	23
2003	742	43	462	27	62	1463	1071	73	8822	2418	27
2004	946	53	545	30	58	2006	1479	74	13,200	3337	25
2005	1048	62	610	36	58	2381	1795	75	14,707	3951	27
2006	863	52	532	32	62	1964	1538	78	11,639	3485	30

Na: not available data.

**Table 3:** Sickness absence in a shipyard industry in Greece in 1999**–**2006, presented by year using different measures.

Year	Mean number of sick-leave days per employee ( without weekends or holidays)	Employment time loss due to sick-leaves (%) ^1^	Frequency of sickness absence ^2^	Cumulative incidence of sickness absence (%) ^3^	Sick-leave rate corrected ^4^ (%)
1999	5.2	0.94	60.2	35.7	2.24
2000	5.1	1.01	58.9	38.0	2.20
2001	4.6	1.09	53.2	41.1	1.98
2002	6.5	1.04	89.8	47.0	2.80
2003	5.1	0.75	90.2	43.1	2.20
2004	7.3	1.13	105.5	52.6	3.15
2005	8.7	1.11	147.3	61.6	3.75
2006	7.1	0.89	127.5	52.3	3.06

^1^ Employment time loss due to sick-leaves (in %) = number of employees with at least one spell of absence x mean duration of absence (not including weekends)/number of employees × 232 working days (in days); ^2^ Frequency of sickness absence (or Sick-leave frequency) = number of sick-leave spells/year/number of employees; ^3^ Cumulative incidence of sickness absence = number of people with at least one new sick-leave spell/number of employee; ^4^ Sick-leave rate (or sickness absenteeism rate; corrected for weekends) = total number of sick-leave days/(total number of employees × number of working days (232)).

Several reasons could explain the low sickness absence rate in Greece, as well as the diversity that exists across countries. Osterkamp and Röhn have reported several of these reasons in 2007; many applied in Greek case [7]. Firstly, the low compensation rate (50%) in Greece, which has been proven to play a key role [23,24]. Countries like Sweden where social insurance schemes provided generous sickness absence compensation have been shown to have high levels of sickness absence [24,25]. A second reason is the difference in unemployment rates. The unemployment rate has been rather high (about 10% of the population) in Greece during the study period compared to most countries in the EU according to Eurostat [26,27].

The employment rate in Greece during the study period according to the Fourth European Survey was 59.6%, one of the lowest in the European Union. Retirement pensions in the private sector typically start at 60 after 35 years of employment but early, nondisabled retirees included men at age 58 years who completed 35 years of work and those who work in hazardous occupations (“heavy and unhealthy jobs”) at ages 55 or 53 years for full or partial retirement, respectively [28]. In theory higher unemployment rates mean greater difficulties in finding a new job in case of dismissal, and therefore greater effort by the employee to stay at work even with impaired health (*i.e.*, “presenteeism”) but the real effects of these parameters (unemployment or employment rate and retirement schemes) on sick leave duration are not well documented [7].

Another parameter could be the differences in the educational level among employees. According to the 4^th^ European Working Conditions Survey [18] around 35% of employees in Greece are of primary or lower secondary education, one of the highest proportion in the EU, and less than 20% have received any special training. In addition, Greek employees reported one of the lowest levels of autonomy at work [18,29] which is possibly related to supervision and motivation. It is important to investigate the role of the psychosocial working environment on sickness absence. Paradoxically, work load, which is considered to affect sick leave rate, is reported as heavier in Greece and it is related to a lengthy working week (over 40 h) [18,19], with 40% of employees (including self-employed) working even more.

Furthermore, diversity in health-ill patterns among populations might partly explain differences in sickness absence rates. However, it is not very plausible to explain more than a small fraction since morbidity patterns did not vary so much at least in industrialized countries [7]. Several reports have shown higher morbidity levels among Greek employees or at least similar to the rest European population [25,30] while according to the 4^th^ European Working Conditions Survey [18] Greek employees are not only exposed to occupational hazards to a greater extent compared to other Europeans, but they report far more frequently that are not very satisfied with their work. These facts are in contrast with the low sick leave rate in Greece, which besides the low compensation rate is hardly excusable and remains an interesting case to be explored, perhaps in the direction of personal, work and socio-cultural related factors. For example, the increased family support in Greek society [31] enables employees to keep working, even under moderate health conditions [29,31] or the management toleration in some unofficial (and not recorded) sick leaves. 

We have to acknowledge that the results of this study cannot be applied to the public sector which it has structural differences and data on sickness absence are not available. Even though shipyard data cannot be considered as representative of private employment in Greece, the comparable indexes were very much alike with the national data from the major insurance scheme (IKA). Although higher than previously recorded the fact remains; Greece ranks last or first in the list with six or less days lost annually per employee in private sector. 

## 3. Experimental Section

In this study, we have described and compared time series of data on sickness absence originated by (i) shipyard employees; and (ii) the largest social insurance fund in Greece, which covers the majority (70–90%) of employees in the private sector. 

### 3.1. Shipyard Industry Data

Between 1999 and 2006, 1850–1900 employees (on average) of a shipyard company have been monitored for sickness absences each year during the study period by the Occupational Health Department (OHD). All permanent employees were included in the study, including metal workers (e.g., platters, fitters, and pipe fitters), welders, drivers/crane operators, carpenters, electricians, sandblasters/painters, and a variety of other technicians, production workers and support staff. Accountants, designers, secretaries, telephone operators, computer experts, managers, engineers and other professionals were also included. 

For each subject information on the frequency and duration of spells of sickness absence was retrieved from medical certifications issued by physicians (private or from the Social Insurance Institute). In order to verify the cause of absence, on return to work medical staff interviewed the worker and categorized sickness absence into 13 disease groups. A return to full duty work of at least 1 day was needed to consider the next episode of sick leave as a new event *i.e.*, a sick-leave spell (episode of sickness absence). Due to the very small percentage (<0.2%) of ongoing sick-leave spells over the change of years (December–January) combined with the extremely low proportion of long term sick-leaves (>14 days), we decided to analyze each year separately by assigning sick leaves in the year that they have started. In addition, data on the compensated days due to sickness absence data were provided by the Human Resources and Accounts Department and utilized to increase validity (tracing missing sick leaves and for confirmation).

Based on the field data the following indicators of sickness absence were calculated:

(i) Number of sick-leave days/employee = mean number of sick-leave days/year/number of employees
(ii) Frequency of sickness absence (or Sick-leave frequency) = number of sick-leave spells/year/number of employees
(iii) Cumulative incidence of sickness absence = number of people with at least one new sick-leave spell/year/number of employee
(iv) Mean duration of sick-leave spells = all sick-leave days/number of sick-leave spells
(v) Employment time loss due to sick-leaves (in %) = number of employees with at least one spell of absence x mean duration of absence (not including weekends)/number of employees × 232 working days
(vi) Sick-leave rate [or sickness absenteeism rate, (in %)] = total number of sick-leave days/[total number of employees x number of working days (232)]

The study population each year was calculated as the mean of the five corresponding values of the total number of employees, which it was updated every three months.

### 3.2. Social Insurance Institute (IKA) Data

IKA is the largest statutory social insurance fund in Greece and covers the majority (70**–**90%) of employees in the private sector, white and blue-collar workers [11,32]. During the study period, IKA insured in average of 1.8−2 million employees (see Table 1), another 5.5 million were covered as dependent family members and 300,000 as pensioners. Insured are mostly men (52%; around 60% in industry) and occupied as office workers (22.8%), as employees in Sales, Services, Mining, Construction, Manufacturing & Transport (22.6%), as service, shop and market sales workers (15%), as technicians, craft and related workers (19%), *etc*. (for further information on the male population distribution according to ISCO-88, Revised International Standard Classification see [33]). 

Data regarding the total number of employees insured at IKA between 1987 and 2006, and compensated sick-leave days (sick-leave days for which they received salary compensation from IKA), were retrieved from the annual reports published online [34]. Information regarding the period 1987–1997 for which no data were published online by IKA, were retrieved from a PhD thesis [11]. Since compensation starts from the fourth day of sick leave, short sick-leave spells (<4 days) are not compensated, and therefore not recorded. In order to approximate the real total sick-leave days, firstly (i) we have added the first 3 days of each recorded spell that were not compensated; considering that 20% of insured employees had at least one spell longer than 3 days, and then (ii) multiplying by a factor *y* = 1.33 in order to take into account the not recorded short spells (<4 days) extrapolating the relative data from the shipyard cohort (at least 1 out of 4 spells was short; less than 4 days). This approximation has also been suggested in the PhD thesis [11], but it has not been used in reports. 

Finally, we calculate sick leave rates by using as an annual mean the 232 days per employee. Statistical Package for Social Sciences (SPSS) program-version 17.0 was used for data entry and analysis. 

## 4. Conclusions

The low level of sickness absences in private sector in Greece was confirmed in our study, although it was found higher than the suggested in previous international studies. Sick leave rate was 2.5% and the total duration per employee was 5.8 days/year. Interestingly, short spells (<4 days) of sickness absences accounted at least for 25% of the total duration which currently is not recorded in national statistics. In the 20-years national data, results have shown a 7-year wave in sickness absence indexes (a decrease the period 1991–1997 and an increase in 1998–2004) combined with a small yet significant decline as a general trend. These observations deserve detailed attention and could only partly be attributed to the compensation and unemployment rates in Greece, while other factors should be explored in field studies whereas sick-leaves should always be monitored and evaluated as an assessment tool of the working environment.
